# The Three-Dimensional Growth of Traumatic Intracerebral Hemorrhage in Patients with Abnormal Coagulation

**DOI:** 10.1055/a-2737-7527

**Published:** 2026-02-27

**Authors:** Jakob Rossmann, Johannes Falter, Julius Höhne, Christoph Hohenberger, Lisa Bründl, Nils Ole Schmidt, Karl Michael Schebesch, Sylvia Bele

**Affiliations:** 1 Department of Neurosurgery, Paracelsus Medical University Nuremberg, Nuremberg, Germany; 2 Department of Neurosurgery, University Medical Center Regensburg, Regensburg, Germany

**Keywords:** traumatic intracerebral hemorrhage, coagulation, volumetry

## Abstract

**Background and Purpose:**

The goal of the present study was to compare the volumetric three-dimensional growth of traumatic intracerebral hemorrhage (tICH) in patients with and without abnormal coagulation and to question the necessity to perform repeated CT scans in all those patients.

**Methods:**

We retrospectively analyzed CT scans from 50 patients with tICH. Abnormal coagulation was defined by the results of standard coagulation tests at admission, including Factor XIII. The three-dimensional size of the hemorrhage was measured at admission, within 48 hours, and 2 weeks.

**Results:**

Growth of the tICH was detected in 56% of the patients. In the group with normal coagulation (n = 30 patients), growth could only be detected in 10 (33.34%) patients, whereas in the abnormal coagulation group, volume increase occurred in 18 of 20 patients (90%). The mean growth was 3.46 mL (95% CI:±2.99 mL) and varied from 0.1 mL (95% CI: ±1.57) in the normal coagulation group to 8.52 mL (95% CI: ±6.67 mL) in the coagulation disorder group.

**Conclusion:**

This study demonstrates the need to perform repeated CT scans in patients with coagulation disorders since patients with tICH and coagulation abnormalities are likely to experience substantial growth of the hemorrhage.

## Background and Purpose

Traumatic brain injury (TBI) is still the most common reason for disability and death in young adults in industrialized countries. 1 But, in recent years, a growing number of elderly patients suffering from TBI with lethal outcomes was registered by the “Federal Statistical Office” in Germany in 2022. 2 In addition, the German TraumaRegister revealed an increase in the age of patients suffering from an isolated TBI, mainly due to falls below 3 m within the home area. 3 As patients get older, there is an increasing number of comorbidities, for example, heart disease, requiring anticoagulant medication, potentially leading to complications following TBI. Thus, it is not surprising that the mean age of patients dying from TBI increased from 52 years in 1998 to 75 years in 2021. 4

One risk of neurological deterioration following TBI is the potential growth of contusion size in the first 12 to 24 hours following TBI. In recent two-dimensional studies, the amount of hemorrhage growth in TBI patients ranged from 38% to 65%. 5 6 7 8

Thus, for patients with severe TBI, who are often not neurologically assessable, the decision to perform regular control CT scans is mostly based on these numbers, leading to up to five CT scans in TBI patients. 9 In 1992, Stein et al described a correlation between coagulation and progressive hemorrhagic injury, making the decision for additional CT scans even more complex. 10

For patients with mild TBI, physicians can decide to perform CT scans according to the New Orleans or the Canadian CT Rule, which considers clinical appearance, age, and patient history. 11 12

This study aims to help physicians make decisions about repeated CT scans in patients with traumatic intracerebral hemorrhage (tICH) by examining the frequency of volume growth of tICH in patients with and without coagulation disorder.

## Methods

We performed a retrospective single-center analysis of patients of all ages admitted to our emergency room (ER) between January 1, 2010, and December 31, 2016. We only included patients with tICH in the initial head CT, after other reasons for bleeding had been ruled out by questioning the patient, relatives, or the persons who saw the incident. Only patients with at least one control CT scan within 48 hours (range 6–48 hours) and one more within 2 weeks were included. We excluded all patients with additional intracranial lesions, such as epidural or subdural hematoma, to avoid additional effects on contusion volume. Additional exclusion criteria were absence of trauma, typical hypertensive ICH, craniotomy after the initial CT scan, implementation of an intracranial pressure monitoring, or decision for best supportive care therapy after initial diagnosis.

All patients received standard intensive care unit (ICU) care, keeping the systolic blood pressure <150 mm Hg, temperature between 36 °C and 37.5 °C, SO _2_ >95, and if mechanically ventilated, PaO _2_ >80 mm Hg, and PaCO _2_ between 33 and 45 mm Hg. Hypothermia below 35.5 °C was avoided using heating blankets to minimize the influence of body temperature on coagulation.

We determined demographics like age, sex, admission laboratory results, drug use, and coagulation disease from the electronic patient records.

The ethics board of the University of Regensburg granted ethical approval (16-160-0122). All research was conducted according to the Declaration of Helsinki. Informed consent was waived due to the strict retrospective form of this study.

### Head Computed Tomography

Initial head CT scans (CT 1) were either performed in the transferring clinic or on admission to our ER. Patients suffering from TBI with pathological CT scans or neurologic symptoms were admitted to our neurosurgical ICU for further evaluation. Following the recommendation in previous studies, a control CT scan (CT 2) was performed in all included patients within 48 hours and 2 weeks after trauma (CT 3). 9 12 13 14 The timeframe of the first control CT scan ranged between 6 and 48 hours, depending on the patient's neurologic exam or clinical deterioration. The volume of tICH was measured three-dimensionally using IPlanNet 3.0.0 (Brainlab AG, Munich, Germany).

### Coagulation Analysis

Our standard laboratory tests in the ER on admission included international normalized ratio (INR), partial thromboplastin time (PTT), thrombocytes, Factor XIII, and fibrinogen in trauma patients. The patient's history was also checked for medication with anticoagulants.

### Statistical Analysis

The statistical analysis was performed using IBM SPSS Statistics 25. Data were tested for normal distribution with the Kolmogorov–Smirnov test, and the significance was tested using the *t* -test for independent data. Non-normal data were tested using the Mann–Whitney U test for independent variables. Significance was set at *p* ≤ 0.05.

## Results

We analyzed data from 372 patients diagnosed with isolated TBI. Out of these, we identified 50 patients, 40 males and 10 females, between 6 and 91 years, fulfilling our inclusion criteria. Baseline patient characteristics are shown in Table 1 . The average age was 52.2 years. There was no difference in age or sex distribution between the two groups.

**Table 1:** Baseline characteristics of included patients

	All	Normal coagulation	Abnormal coagulation
*n*	50	30	20
Male	40 (80%)	24 (80%)	16 (80%)
Female	10 (20%)	6 (20%)	4 (20%)
Age (years; mean)	52.2	52.1	52.4

**Fig. 1:**
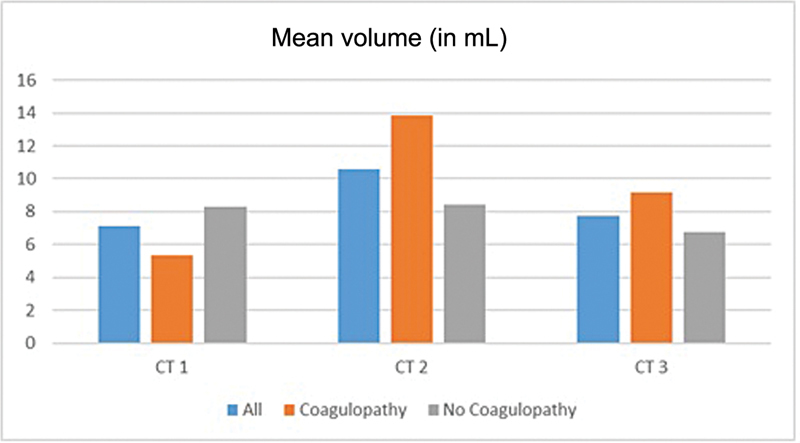
Mean volume of the traumatic intracerebral hemorrhage in the computerized tomography scans in mL for all patients, patients with coagulopathy and patients without coagulopathy.

**Fig. 2:**
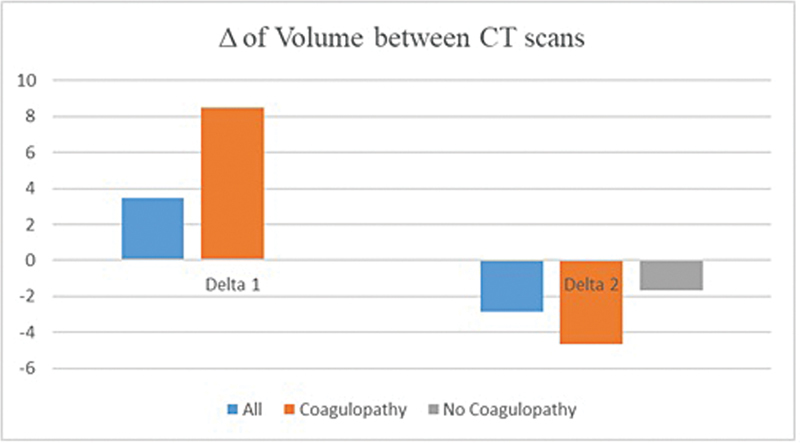
Difference in mean volume of the traumatic intracerebral hemorrhage between first and second computerized tomography (CT) (Delta 1) and second and third CT scan (Delta 2; in mL) for all patients, patients with coagulopathy, and patients without coagulopathy.

Table 2 and Figures 1 and 2 show our volumetry results. TICH growth was seen in 28 patients (56%). The initial volume was 7.12 (95% CI: 5.27–8.97) mL with a standard deviation of 6.70 mL.

**Table 2:** Results of volumetry showing amount of growing and shrinking traumatic intracerebral hemorrhage (tICH) as well as mean values of volume of computerized tomography (CT) scans at all time points and calculated values for the mean differences, all in mL

	All	Normal coagulation	Abnormal coagulation
Growing tICH	28 (56%)	10 (33.34%)	18 (90%)
Shrinking tICH	22 (44%)	20 (66.67%)	2 (10%)
Volume in CT 1 (in mL)	7.12	8.30	5.34
Delta 1 (in mL)	3.46	0.10	8.52
Volume in CT 2 (in mL)	10.58	8.40	13.86
Delta 2 (in mL)	−2.86	−1.67	−4.66
Volume in CT3 (in mL)	7.72	6.73	9.20

The average growth between CT 1 and CT 2 was 3.46 (95% CI: 0.47–6.45) mL, with an average volume of 10.58 (95% CI: 7.26–13.90) mL in CT 2.

The difference between CT 2 and CT 3 was −2.86 (95% CI: −4.29–1.43) mL, leading to an average volume of 7.72 (95% CI: 5.16–10.28) mL in CT 3.

Normal coagulation at admission was seen in 30 patients. Only 10 patients (33.3%) showed growing tICH. The initial volume was 8.3 (95% CI: 5.64–10.96) mL. Average ICH growth between CT 1 and CT 2 was 0.1 (95% CI: −1.47–1.67) mL. Volume in CT 2 was 8.4 (95% CI: 5.36–11.46) mL.

Between CT 2 and CT 3, the volume decreased by 1.67 (95% CI: −2.37–0.97) mL, leading to an average volume of 6.73 (95% CI: 3.52–9.94) mL in CT 3.

Of the 20 patients with coagulopathy, 18 (90%) showed an increase in volume from 5.34 (95% CI: 3.05–7.63) mL in the CT 1 to 13.86 (95% CI: 6.99–20.73) mL in the CT 2. The difference between CT 1 and CT 2 was 8.52 (95% CI: 1.85–15.19) mL. Between CT 2 and CT 3, a volume reduction of 4.66 (95% CI: −8.01–1.31) mL was seen. The volume in the CT 3 was 9.20 (95% CI: 4.86–13.54) mL.

The results showed a significantly higher risk of increasing tICH volume in patients with coagulopathy ( *p* < 0.001) as well as a more significant increase in ICH volume between the first two CT scans ( *p* = 0.001).

## Discussion

In the present study, we evaluated changes in hematoma volume of tICH during the first 2 weeks after the accident, using three-dimensional measurement in the initial CT scan, within 48 hours, and within 2 weeks to evaluate the importance of the control CT scan in TBI. Patients were divided into two groups, one without and one with coagulopathy. We could show an increase in hematoma volume in approximately 90% of patients with coagulopathy and in 56% of the patients with normal coagulation. Thus, more than half of all patients suffering from TBI with tICH are at risk of developing an increase of the hematoma size, possibly leading to neurologic deterioration. These numbers are in accordance with former studies and raise the question about the correct time point and repetition of CT scans, especially in patients, which cannot be tested neurologically. 5 6 7 8

### Guidelines and Neurological Exam

Unfortunately, the actual guidelines do not include clear recommendations concerning the timeframe and number of control CT scans following tICH. In mild TBI without intracerebral contusions or assessable patients, we believe that repeated neurological exams are most important to decide if a second CT scan is needed. In their landmark textbook, Greenberg supports a repeated CT scan after a decline in Glasgow Coma Scale by two or more points, as proposed by Teasdale and Jennett. 15 16 However, neurologic deterioration might occur unrecognized in patients with severe TBI, being sedated. In consequence, clinicians might not be able to use repeated neurologic testing for decision making whether an additional scan is needed, unless ICP measurement is installed.

ICP monitoring is often limited to high-income countries. In addition, impaired coagulation carries an increased risk of additional ICH following the implementation of the ICP probe, which might favor control CT scans instead. Even though ICP monitoring is recommended in the TBI guidelines, no level 1 evidence supports ICP-guided therapy, especially with regard to patient outcome. 17 18

### Repeated CT Scans

Since frequent CT scans can might harm young patients, the present study was performed to examine if all tICHs grow within 48 hours and if routine CT controls within this time are essential. 19

In the second step, we analyzed the difference in frequency of ICH growth over time and growth volume in patients with normal coagulation and with coagulopathy to better understand which patients might benefit from regular control CT scans.

We saw growth of the initial contusion in 56% of our patients. This is similar to prior studies, in which volume increases ranging from 38 to 65% 5 6 7 8 was reported. That supports the recommendation for at least one control CT scan within the first 48 hours following TBI, especially if the patients are deteriorating or cannot be assessed neurologically. The exact time point for a control CT scan cannot be determined by the data of the present study due to the inconsistent time points of the second CT scans.

In patients without comorbidity or coagulopathy under strict clinical observation, a control CT scan can be omitted if the patients remain in good neurological condition: An ICH volume increase was found in only 33% of patients with normal coagulation and that volume increase consisted of only 0.1 mL at average.

In contrast, we noticed a growth in ICH volume in 90% of patients with abnormal coagulation. In addition, the increase in lesion size was significantly higher than in the patients with regular coagulation. These findings are in accordance with previous studies showing that coagulopathy increases the risk for progressive hemorrhage following TBI. 20

An interesting finding was that patients with coagulopathy had a smaller initial ICH volume than the patient with normal coagulation, combined with a more significant increase in ICH volume over time. This emphasizes the need for control CT scans in this group.

### Coagulation in Traumatic Brain Injury

The underlying pathophysiological mechanism leading to a secondary increase in hematoma volume is damage to vessels at the edge of the hemorrhage. This leads to a dysfunction of the vessel, petechial bleeding, and a leaking blood–brain barrier. This is followed by cytotoxic edema with necrosis, the risk of rebleeding and, as a consequence, hematoma growth. 21

This risk for rebleeding is increased by dysfunctional coagulation of whichever etiology. In addition, TBI itself can alter coagulation in different ways, like changes in Factor XIII. 22 23 24 Several groups showed decreased Factor XIII activity in up to 20% of TBI patients, although the exact mechanisms remain unclear. Congenital factors, increased turnover in hyperfibrinolysis, decreased synthesis due to liver disease, and consumption due to trauma were postulated. 24 25 26

Also, several works pointed toward the intake of anticoagulants as an important factor in progressing ICH and worse outcomes following trauma. 27 28

Other authors have differentiated between coagulopathy through the intake of anticoagulants and trauma-induced coagulopathy. 20 This difference was not crucial for this study, since the aim was to give recommendations about control CT scans. Thus, we did not differentiate between the underlying reasons for coagulopathy in this paper, but we will follow up on this point in a larger patient group.

In our patient group, three patients under 18 years were included, two of whom were 16 and 17 years old, and one was 6 years old. We did not exclude those patients since the two older ones were close to adulthood. The foremost intention of the study was to investigate the potential volume increase of contusions, not the underlying conditions, and the plausibility of control CT scans in the routine management of TBI patients.

## Limitations

The limitations of this work are the retrospective study design and the limited number of patients included, as well as the inclusion of pediatric patients, making generalization of these data difficult. Still, to the best of our knowledge, there have not been any data on three-dimensional changes in tICH volume, excluding other intracranial pathologies.

## Conclusion

In conclusion, our data show the need for control CT scans in patients with tICH and abnormal coagulation. Future research should aim to investigate and reverse the different reasons for coagulopathy to improve the outcomes for those patients.
